# Multi-Objective Optimal Trajectory Planning for Robotic Arms Using Deep Reinforcement Learning

**DOI:** 10.3390/s23135974

**Published:** 2023-06-27

**Authors:** Shaobo Zhang, Qinxiang Xia, Mingxing Chen, Sizhu Cheng

**Affiliations:** 1 School of Mechanical & Automotive Engineering, South China University of Technology, Guangzhou 510641, China; 2 Zhuhai Gree Precision Mold Co., Ltd., Zhuhai 519070, China; 3 Aircraft Maintenance Engineering School, Guangzhou Civil Aviation College, Guangzhou 510403, China

**Keywords:** trajectory planning, deep reinforcement learning, multi-objective optimization, decaying episode mechanism

## Abstract

This study investigated the trajectory-planning problem of a six-axis robotic arm based on deep reinforcement learning. Taking into account several characteristics of robot motion, a multi-objective optimization approach is proposed, which was based on the motivations of deep reinforcement learning and optimal planning. The optimal trajectory was considered with respect to multiple objectives, aiming to minimize factors such as accuracy, energy consumption, and smoothness. The multiple objectives were integrated into the reinforcement learning environment to achieve the desired trajectory. Based on forward and inverse kinematics, the joint angles and Cartesian coordinates were used as the input parameters, while the joint angle estimation served as the output. To enable the environment to rapidly find more-efficient solutions, the decaying episode mechanism was employed throughout the training process. The distribution of the trajectory points was improved in terms of uniformity and smoothness, which greatly contributed to the optimization of the robotic arm’s trajectory. The proposed method demonstrated its effectiveness in comparison with the RRT algorithm, as evidenced by the simulations and physical experiments.

## 1. Introduction

Robotic arms possess attributes such as operational flexibility and heightened safety, enabling them to supplant human labor in executing a wide range of intricate and repetitive tasks. These robots have found extensive applications in sectors such as manufacturing and logistics. Motion planning is a critical aspect that ensures the successful completion of diverse operational tasks by robots. Presently, motion planning research within the realm of control systems has emerged as a focal point in the field of robotics. The motion planning problem can be bifurcated into two components: path planning and trajectory planning. The primary distinction between these components lies in the temporal dimension. Trajectory planning entails the application of temporal laws to the path devised by the path planner [1]. The objective of this process is to facilitate the completion of tasks by the robot within a specified time frame, while adhering to a multitude of constraints. These constraints include ensuring a smooth trajectory, preventing the breach of the robot’s physical limitations, and avoiding collision or contact incidents, among others.

Swarm intelligence optimization methods demonstrate a miraculous ability to manage complex environmental changes, facilitating efficient trajectory planning through autonomous global optimal solution searches, adaptive parameter adjustments, and rapid convergence. The application of these techniques to address trajectory tracking challenges has garnered increasing attention, with examples including genetic algorithms [2,3], particle swarm algorithms [4], and ant colony algorithms [5]. An adaptive elite genetic algorithm incorporating singularity avoidance has been employed for online time-optimal trajectory planning in Cartesian space [2]. To address the path constraint problem in trajectory planning, a time-optimal trajectory-planning method for robotic arms has been proposed, which simultaneously searches for the optimal path [3]. Particle swarm optimization has emerged as a prevalent optimization technique, utilized in robot trajectory planning to guide practical industrial production applications [4]. The current century has seen the development of numerous exceptional bionic-inspired optimization algorithms, such as artificial bee colony algorithms [5], whale algorithms [6], and hybrid algorithms [7,8]. Although these algorithms exhibit impressive performance in resolving trajectory-tracking challenges, their extensive calculation times in dynamic environments may result in unstable optimal solution computations. Recently, the learning-based theories have attracted significant attention in addressing motion-planning issues, owing to their outstanding adaptability to complex problems [9]. Notable examples include deep learning [10,11] and reinforcement learning [12,13]. The learning-based approach employs a machine learning strategy, utilizing data-driven solutions to eliminate dependence on intricate models and reduce the complexity of devising trajectory-planning methods. In robot trajectory planning, position coordinates function as the input parameters, while the joint angles serve as the output. Converged neural network parameters are obtained through sample-based training, with the learning strategy focused on estimating the solution. As the number of samples increases, the final results exhibit progressive improvement [11]. In dynamic environments, traditional offline planning methods become inapplicable when a space robot is in motion. A trajectory-planning strategy based on reinforcement learning enables the robot to rapidly adjust its executed actions, rendering this method suitable for real-time applications [13].

Time-optimal trajectory planning has been extensively investigated [14]. The fundamental concept involves determining the time points of the optimal trajectory within the planning period using optimization algorithms under constraints such as velocity, acceleration, and jerk. Moreover, energy consumption has emerged as a crucial reference factor in trajectory planning [15]. Multi-objective trajectory-planning problems are progressively supplanting single optimal problems, becoming the predominant focus of industrial robot trajectory-planning research. Since intense pulses can induce vibrations in robotic arm motion, joint pulses represent a significant consideration in the trajectory-planning research of robotic arm multi-objective optimization. Liu [16] introduced a trajectory competition multi-objective particle swarm optimization algorithm, addressing cooperative robot trajectory planning from the three aspects of time, energy, and pulse optimization. Hou [17] proposed an innovative time–energy consumption optimization trajectory-planning method for dynamic identification. Rout [18] employed a multi-objective ant lion optimization technique to obtain the optimal trajectory by minimizing the time–torque ratio. Huang [19] drew on the elite non-dominated sorting genetic algorithm, put forward a time-pulse comprehensive optimal-trajectory-planning method for industrial robots. Trajectories generated through multi-objective optimization ensure that robots can attain relatively optimal performance when reaching the same location. However, traditional six-axis robotic arm trajectory-planning methods may encounter challenges in fulfilling multi-objective optimization due to their reliance on numerical optimization, which necessitates precise mathematical models and adherence to the robot’s physical constraints.

To attain more-efficient and -adaptive trajectory planning, this paper presents a six-axis robotic-arm-trajectory-planning method based on deep reinforcement learning. Compared with the existing work, three contributions are presented:

(1) This approach achieves multi-objective optimization through online learning and real-time strategy adjustments, encompassing the comprehensive considerations of accuracy, smoothness, and energy consumption.

(2) In the training process, a decaying episode mechanism was introduced to help find the feasible solution faster, which makes the path planning shorter to help the subsequent trajectory optimize the ground difference in more detail.

(3) By leveraging the properties of reinforcement learning, a trajectory-planning strategy that optimally balances various performance indicators can be identified without depending on precise mathematical models and physical constraints.

The remainder of this paper is organized as follows. Section 2 shows the kinematics and dynamics of the manipulator. Section 3 details the multi-objective optimization problem. Section 4 presents the proposed RL-based multi-objective optimal trajectory-planning method, along with a detailed account of the reward function design. Section 5 offers the simulation, and physical experiments are shown. Section 6 concludes this work.

## 2. Kinematics and Dynamics of Manipulator

**Figure 1:**
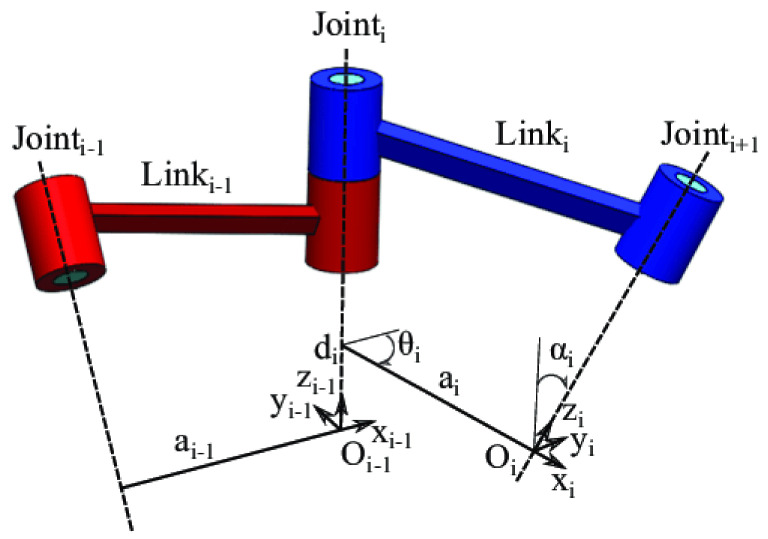
Representation of the Denavit–Hartenberg parameters.

The kinematics of a manipulator represent its motion characteristics, exclusive of mass, inertia, and force considerations. To accurately control a robot’s position, it is essential to construct its kinematic model. Each link of the robot can be characterized by four kinematic parameters, with two describing the link itself and the other two representing the connection relationship between the links. The method in which these kinematic parameters express the kinematic relationship of the robot mechanism is commonly referred to as the Denavit–Hartenberg (DH) method [20], and the Denavit–Hartenberg parameters [21] are shown in Figure 1.

For a six-degree-of-freedom manipulator, the transformation formula for the connecting rod, represented from the base to the end effector, can be obtained by articulating the parameter configuration in the form of a homogeneous transformation matrix, $q=[q_{1},q_{2},...,q_{n}]$:

(1) $${}_{E}^{0}T\left(q\right)=\left(\overset{6}{\underset{i=1}{\Pi }}{}_{i}^{i-1}T\left(q_{i}\right)\right)\cdot {}_{E}^{6}T$$

where ${}_{i}^{i-1}T\left(q_{i}\right)$ is defined as:

(2) $$T\left(q_{i}\right)=\left[\begin{array}{cccc} \cos\theta_{i} & \sin\theta_{i} & 0 & a_{i}\cos\theta_{i}\\ -\sin\theta_{i}\cos\alpha_{i} & \cos\theta_{i}\cos\alpha_{i} & \sin\alpha_{i} & a_{i}\sin\theta_{i}\\ \sin\theta_{i}\sin\alpha_{i} & \sin\alpha_{i} & \cos\alpha_{i} & d_{i}\\ 0 & 0 & 0 & 1 \end{array}\right]$$

In accordance with the forward kinematics of the manipulator as indicated in Equation (1), the configuration space is expressed. Owing to the task-specific configuration of the manipulator’s end effectors, it is often necessary to infer the particular configuration space based on the position space. This process, referred to as inverse kinematics, enables precise motion control for each joint of the manipulator according to the angle. In inverse kinematics, the desired workspace displacement $\dot{x}$ and the initially unknown configuration space displacement $\dot{q}$ are determined:

(3) $$\dot{x}=J\left(q\right)\cdot \dot{q}$$

where J(q) is the Jacobian matrix. The forward kinematics and inverse kinematics of the manipulator are solved, and the optimal trajectory planning of the manipulator based on the reinforcement learning method is realized. The M D-H method [22] is different from the D-H method, which has different connecting rod frames and parameter settings, such as the following:

(4) $${}_{i}^{i-1}T\left(q_{i},q_{i-1}\right)=\left[\begin{array}{cccc} \cos\theta_{i} & -\sin\theta_{i} & 0 & a_{i-1}\\ \sin\theta_{i}\cos\alpha_{i-1} & \cos\theta_{i}\cos\alpha_{i-1} & -\sin\alpha_{i-1} & -d_{i}\sin\alpha_{i-1}\\ \sin\theta_{i}\sin\alpha_{i-1} & \cos\theta_{i}\sin\alpha_{i-1} & \cos\alpha_{i-1} & d_{i}\cos\alpha_{i-1}\\ 0 & 0 & 0 & 1 \end{array}\right]$$

## 3. Multi-Objective Optimization Problem

In this work, we used a deep reinforcement learning (DRL) approach for the trajectory planning of a six-degree-of-freedom manipulator. We aimed to improve the accuracy, enhance the motion smoothness, and minimize the energy consumption. We formulated these goals into a multi-objective optimization problem that our DRL algorithm resolves. Specifically, we prioritized accuracy for precise trajectory tracking, smoothness to reduce joint-impact-induced vibrations, and energy efficiency to decrease overall system consumption.

The trajectory-planning problem can be transformed into a multi-objective optimization problem as:

(5) $$\begin{array}{ccc} F & =\min\left(\sum_{i=1}^{n}\lambda_{i}f_{i}\left(\theta \right)\right)\;\; & s.t.\;h_{ij}\left(\theta \right)\geq 0,\;i,j\in 0,1,\cdots ,n \end{array}$$

where $\theta =[\theta_{1},\theta_{2},\theta_{3},\theta_{4},\theta_{5},\theta_{6}]$ and the weight coefficient of the cost function $f_{i}\left(\theta \right)$, denoted as $\lambda_{i}\in \left[0,1\right]$, represents the *j*-th constraint corresponding to the function. $h_{ij}\left(\theta \right)$ represents the *j*-th constraint condition for the *i*-th joint of the robot. This consideration incorporates limitations related to the joint angle, joint velocity, and joint acceleration.

The requirements of planning trajectory accuracy, trajectory smoothing, and minimum energy consumption were considered. The corresponding cost function is defined as follows:

(1) Accuracy:

This function is mainly concerned with the position and attitude error of the end effector when the manipulator executes the trajectory. The accuracy cost function is defined by calculating the distance between the desired position and the current position of the end effector:

(6) $$f_{a}\left(\theta \right)={∥p_{d}-p∥}^{2}$$

$p_{d}=\left[x_{d},y_{d},z_{d}\right]$ and p=[x,y,z] denote the three-dimensional coordinate positions of the end effectors for the desired trajectory and the actual trajectory, respectively, within the Euclidean space.

(2) Smoothness:

To achieve a smooth trajectory and reduce the vibrational impact caused by the motion of each joint of the manipulator, the smoothness cost function is formulated as follows:

(7) $$f_{s}\left(\theta \right)=\sum_{k=1}^{n}\left(\lambda_{v}{\dot{q}}_{k}^{2}+\lambda_{acc}{\ddot{q}}_{k}^{2}\right)$$

The number of joints is denoted as *n*, and $\lambda_{v}$ and $\lambda_{a}$ are the weight coefficients of the velocity and acceleration, respectively. $\dot{q}$ and $\ddot{q}$ are the velocity and acceleration of the *i*-th joint, respectively.

(3) Energy consumption:

The energy consumption cost function primarily addresses the energy consumption associated with the manipulator’s joint movements. By taking into account the sum of the squared angular changes and joint torques within the discretized unit time, the energy consumption cost function is represented as follows:

(8) $$f_{e}\left(\theta \right)=\sum_{k=1}^{n}{\left(\Delta \theta_{k}\tau_{k}\right)}^{2}$$

Here, Δθ signifies the angular change per unit time, and $\tau_{k}$ represents the torque of the *k*-th joint.

## 4. Trajectory Planning with Reinforcement Learning

### 4.1. PPO

Proximal policy optimization (PPO) represents a significant advancement in the field of reinforcement learning, particularly in addressing the balance between exploration and exploitation in policy-based methods. PPO has been recognized for its ability to maintain the stability of policy updates without sacrificing learning efficiency [23].

Reinforcement learning, as a subset of machine learning, is intrinsically about learning from interaction to achieve optimal behavior. Policy-based methods, including PPO, directly parameterize the policy and make updates to maximize expected return. These methods have an advantage over value-based methods due to their ability to handle continuous action spaces and learn stochastic policies. However, they often suffer from high variance, leading to instability during training.

The PPO algorithm attempts to mitigate this issue by optimizing an objective function designed to keep policy updates close to the current policy:

(9) $$L\left(\theta \right)=E_{s_{t},a_{t}∼\pi_{\theta_{old}}}\left[\frac{\pi_{\theta }\left(a_{t}|s_{t}\right)}{\pi_{\theta_{old}}\left(a_{t}|s_{t}\right)}A^{\pi_{\theta_{old}}}\left(s_{t},a_{t}\right)\right]$$

This objective function maintains the relative advantage of the new policy over the old policy, encouraging the exploration of new behaviors, but penalizing deviations that would significantly alter the current policy.

The cornerstone of PPO is the introduction of a novel surrogate objective function with clipping, which further constrains the policy updates to a neighborhood around the old policy:

(10) $$L^{CLIP}\left(\theta \right)=E_{s_{t},a_{t}∼\pi_{\theta_{old}}}\left[\min\left(\frac{\pi_{\theta }\left(a_{t}|s_{t}\right)}{\pi_{\theta_{old}}\left(a_{t}|s_{t}\right)}A^{\pi_{\theta_{old}}}\left(s_{t},a_{t}\right),\mathrm{clip}\left(\frac{\pi_{\theta }\left(a_{t}|s_{t}\right)}{\pi_{\theta_{old}}\left(a_{t}|s_{t}\right)},1-\epsilon ,1+\epsilon \right)A^{\pi_{\theta_{old}}}\left(s_{t},a_{t}\right)\right)\right]$$

Here, ϵ is a hyperparameter that defines the limit of the policy update clipping. This clipping mechanism ensures modest changes in the policy at each step, promoting stability during the learning process while preventing drastic policy deviations, which could lead to suboptimal solutions or convergence instability.

As the PPO algorithm undergoes iterations and updates, the final trajectory points of the robotic arm incrementally approach the ultimate target point. However, due to the inherent limitations within the algorithm, the points may not fully coincide with the designated target end pose. To address this, after planning using the deep reinforcement learning algorithm, this study incorporated the target end pose point settings into the planning point set, analogous to the planning method employed by the RRT algorithm [24]. Consequently, the trajectory-planning method utilized in this study ultimately forms a closure from the starting point to the end point, enhancing its applicability in real-world scenarios.

### 4.2. Reinforcement Learning Settings

#### 4.2.1. Observation and Action Spaces

Utilizing the PPO reinforcement learning algorithm, the states incorporated into the observation space of the environment encompass the current state $\theta_{start}$, current position $p_{c}$, target state $\theta_{end}$, and target position $p_{d}$ for each of the six joints in the robotic arm, as well as the Euclidean distance $d_{e}$ between the present end pose and the target point. The end of the robotic arm is considered the end effector, culminating in a total of 25-dimensional state variables. Moreover, if the motion state of the robotic arm surpasses its own constraints, the specific joint state exceeding the observation space will be adjusted to its maximum limit value.

The action space is configured to correspond to the speed ${\dot{\theta }}_{k}$ of each joint, and the state of the observation space is updated with every provided action.

#### 4.2.2. Reward Function

The trajectory-planning problem necessitates addressing both temporal considerations and the inherent complexity of the robotic-arm-planning task, thus requiring a meticulously designed reward function. In light of the optimization principles previously discussed and in conjunction with real-world reward requirements, the present study proposes the following reward function $r(s_{t},a_{t})$:

(11) $$r\left(s_{t},a_{t}\right)=r_{a}^{t}+r_{s}^{t}+r_{e}^{t}+r_{ex}^{t}$$

The components $r_{a}^{t}$, $r_{s}^{t}$, and $r_{e}^{t}$ of the reward function pertain to accuracy, smoothness, and energy consumption, respectively. Subsequently, the following optimal constraints can be derived:

(12) $$r_{a}^{t}=-w_{a}\exp(\sigma_{a}f_{a}\left(\theta^{t}\right))$$

(13) $$r_{s}^{t}=-w_{s}\sum_{k=1}^{k}\left(\lambda_{v}{\dot{q}}_{k}^{2}+\lambda_{acc}{\ddot{q}}_{k}^{2}\right)$$

(14) $$r_{e}^{t}=-w_{e}\sum_{k=1}^{k}(\Delta \theta_{k}\tau_{k}{)}^{2}$$

Here, $w_{a}$ and $\sigma_{a}$ represent the weight coefficient of the accuracy reward, while $w_{s}$, $\lambda_{v}$, and $\lambda_{acc}$ are the weight coefficients of the smoothness reward. $w_{e}$ denotes the weight coefficient of the energy consumption reward.

The extra reward $r_{ex}^{t}$ is provided when the current training performance achieves a specified threshold, such as attaining a predetermined minimum distance between the current and desired end point distances.

(15) $$r_{ex}^{t}=\left\{\begin{array}{cc} 10 & \mathrm{if}\;f_{a}\left(\theta^{t}\right)<0.005\\ \frac{10}{1+10f_{a}\left(\theta^{t}\right)} & \mathrm{otherwise} \end{array}\right.$$

### 4.3. Multi-Objective Optimal Trajectory Planning

In a deep-reinforcement-learning-based trajectory-planning approach, the traditional total cost function can be transformed into a corresponding reward function. Through this transformation, the agent engages in continuous learning and iteration, mapping the objective of minimizing the cost function to the objective of maximizing the reward function. The agent is capable of generating the desired trajectory based on the input conditions. To achieve this transformation, it is necessary to convert various measures in the cost function into corresponding rewards. This conversion is accomplished by using the negative value of the cost item as part of the reward function. Thus, during the optimization process, the agent naturally chooses the behavior that minimizes the cost. Next, these rewards are integrated into a comprehensive reward function.

Within the reinforcement learning framework, the agent selects actions according to the current state and adjusts strategies by interacting with the environment. Throughout this process, the agent utilizes the received reward to continuously update the behavior strategy, aiming to maximize the reward while minimizing the cost. By employing the method, the traditional trajectory-planning problem is transformed into an optimization problem based on deep reinforcement learning. In the learning process, the agent gradually adapts to the task requirements in order to generate high-quality trajectories according to the given input conditions. This approach not only optimizes the smoothness, energy consumption, and accuracy of the trajectory, but also enables the introduction of other constraints or objectives as required by the practical application scenario, ensuring that the trajectory planning aligns more closely with the actual needs.

**Figure 2:**
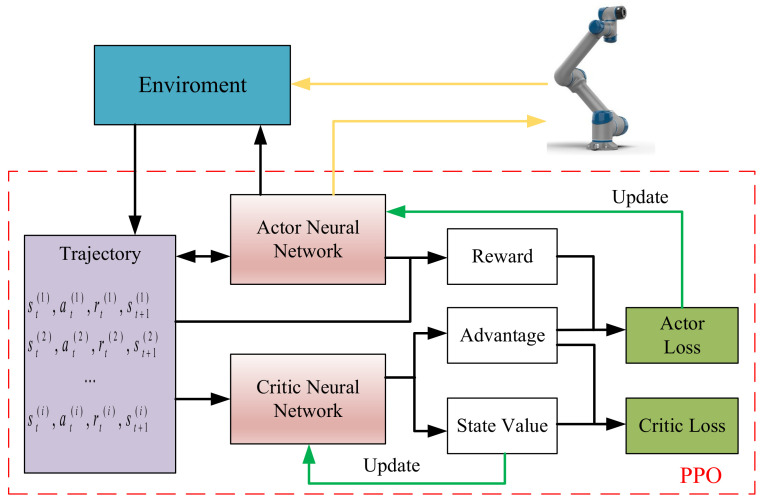
A method framework of 6-dof manipulator trajectory planning based on PPO.

The concept of obtaining optimal trajectory planning through the cost function is transformed into a deep reinforcement learning method, which involves transforming the total cost function into a specific reward function. By minimizing the cost function and mapping it to the reward maximization settings, the agent is able to plan an ideal trajectory based on the input. The overall framework is illustrated in Figure 2.

As depicted in Figure 2, reinforcement learning serves as a critical mechanism in the trajectory planning for a six-axis robotic system. An episode within this context is delineated as a sequential arrangement of states, actions, and rewards that initiates from an initial configuration and culminates upon achieving a predefined terminal condition within the operational environment.

The initial state is a representation of the robot’s configuration, which is articulated as a randomly selected pose, embodying both the positional attributes and potential velocities of each of the six joints in the robot’s structure. Actions are determined on the basis of the current state and are expressed as alterations in the angular positioning of the robot’s joints.

The execution of an action consequently leads to a transition of the robot to a new state, a process that is steered by the robot’s inherent kinematic properties. In tandem with this transition, the robot is accorded a reward, the extent of which is dictated by a composite measure of factors including accuracy in task performance, the energy consumed in the action’s execution, and the smoothness of the movement, thereby ensuring a balanced consideration of performance and efficiency.

The episode reaches its terminus upon the attainment of a goal configuration, indicating the successful completion of a given task. The overarching aim of the reinforcement learning framework is the procurement of an optimal policy—a strategic decision-making blueprint that optimizes the cumulative reward accumulated over the duration of an episode. This is achieved through a continuous, iterative interaction with the environment, allowing the system to learn from its experiences and progressively improve its performance.

The decaying episode mechanism was set up, which automatically sets the step number of one episode to the current step in the current episode when the training accuracy $d_{e}$ reaches a setting value, which will produce a new stable state in the training process, but will not affect the actual performance of trajectory planning. The PPO algorithm of this paper is shown in Algorithm 1.
**Algorithm 1** Proximal policy optimization (PPO) for trajectory planning**Require:** Start $\theta_{start}$, end angle $\theta_{end}$, hyperparameters of actor and critic networks, learning rate α, discount factor γ, generalized advantage estimation (GAE) parameter λ, clipping parameter ϵ, entropy coefficient $c_{ent}$, number of iterations *N*, number of epochs *E*, batch size *B*, episode length *T*, accuracy setting $d_{e}$.
1:**for** n=1,2,…,N **do**2:      Collect *T* time steps transition $\left\{\begin{array}{cccc} s_{t} & a_{t} & r_{t} & s_{t+1} \end{array}\right\}$ using the running policy $\pi_{\theta }$3:      **if** $f_{a}\left(\theta \right)<d_{e}$ **then**4:          $T\leftarrow T_{\mathrm{current}}$5:      **end if**6:      Compute advantage estimates ${\hat{A}}_{t}$7:      **for** e=1,2,…,E **do**8:            Randomly sample a batch of *B* time steps9:            Update the critic network parameters by minimizing the loss:10:          $L\left(\phi \right)=\frac{1}{B}\sum_{t=1}^{B}{\left(V_{\phi }\left(s_{t}\right)-{\hat{R}}_{t}\right)}^{2}$11:          Update the actor network parameters using the PPO-clip objective and the entropy bonus:12:          $\theta_{k+1}=\arg\max_{\theta }\frac{1}{B}\sum_{t=1}^{B}L^{CLIP}\left(\theta \right)+c_{ent}H\left(\pi_{\theta }\left(\cdot |s_{t}\right)\right)$13:          $L^{CLIP}\left(\theta \right)=\min\left(\frac{\pi_{\theta }\left(a_{t}|s_{t}\right)}{\pi_{\theta_{k}}\left(a_{t}|s_{t}\right)}{\hat{A}}_{t},clip\left(\frac{\pi_{\theta }\left(a_{t}|s_{t}\right)}{\pi_{\theta_{k}}\left(a_{t}|s_{t}\right)},1-\epsilon ,1+\epsilon \right){\hat{A}}_{t}\right)$14:      **end for**15:**end for**


## 5. Experiments

### 5.1. Environment Settings

In this study, we employed a six-axis robotic arm with a 5 kg payload capacity, manufactured by SIASUN. The robot manipulator’s kinematic characteristics were described using the modified Denavit–Hartenberg (M D-H) parameters. Table 1 presents the M D-H parameters for each link of the robot, providing a comprehensive overview of the manipulator’s characteristics.

In Table 1, $a_{i}$, $\alpha_{i}$, and $d_{i}$ denote the link length, link twist, and link offset, respectively, while $m_{i}$ and $I_{xx}$, $I_{yy}$, and $I_{zz}$ represent the mass and the moments of inertia of each link, respectively.

In order to validate the efficacy of the proposed algorithm, a series of experiments was conducted using the digital model parameters derived from the physical entity of the Siasun cooperative robot. The constraints of each joint of the manipulator were set as [−ππ], with a particular emphasis on Joint 4, which was constrained by [−8/9π8/9π]. The initial *T* was set as 10, and the reward function weight coefficient $w_{a}=w_{s}=\lambda_{v}=\lambda_{acc}=w_{e}=1,\sigma_{a}=2$, and the accuracy was set as $d_{e}=0.003$.

The training environment for the reinforcement learning process was established using a simulation environment based on the deep learning framework PyTorch and Gym. The hyperparameters employed by the PPO algorithm are presented in Table 2. In order to further demonstrate the algorithm’s effectiveness and adaptability in practical applications, the practical experiments were conducted on the trajectory planned by the proposed method in a collaborative robot.

The paper used two different neural networks, namely the actor network and the critic network, as continuous action spaces. The implementation of the network was based on the PyTorch framework. The structure of the actor and critic networks is shown in Table 3.

### 5.2. Performance Evaluation in Simulation Environment

Assuming the center of the actuator at the end of the robotic arm is the movement point to be considered, set the starting point to $q_{start}=\left[0,0,27\pi /35,7\pi /35,0,0\right]$ and the stopping point to $q_{end}=\left[3,1.5,1.5,2,2,2.5\right]$.

To provide a more-comprehensive evaluation of the proposed methods, this study included an analysis of the bidirectional rapidly-exploring random tree (Bi-RRT) algorithm, an advanced variant of the RRT algorithm. The RRT, a sampling-based motion planning technique, progressively develops a tree structure to navigate the robot’s configuration space. This method demonstrates exceptional effectiveness in high-dimensional spaces and in addressing intricate constraints, making it a pertinent reference point for assessing the performance of the proposed approaches in a scientifically rigorous manner. To maintain consistency in the number of planned trajectory points, the step size of the Bi-RRT method was set to 0.145 m, ensuring efficient planning. Additionally, a bidirectional search method was employed to further optimize the process.

**Figure 3:**
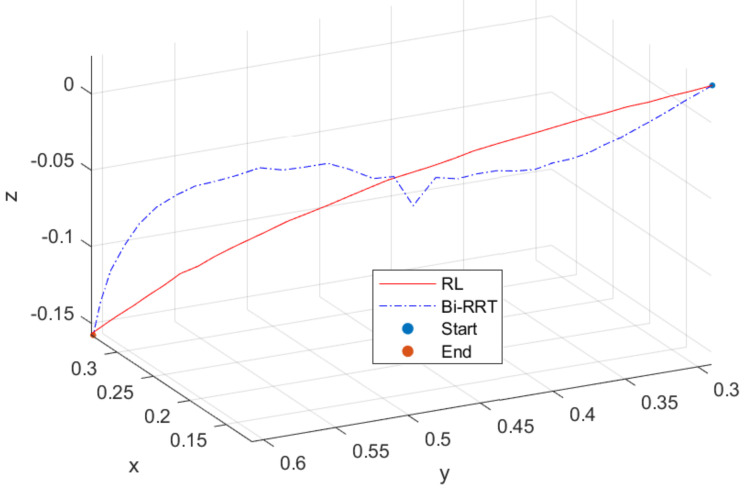
Comparison of trajectory planning between Bi–RRT and RL methods.

Figure 3 presents the trajectories planned through the proposed method and Bi-RRT framework, where the blue dotted line represents the Bi-RRT, the red line signifies the proposed method, yellow dots indicate the starting position of the planned trajectory, and purple dots correspond to the ending position of the plan. As illustrated in Figure 3, both Bi-RRT and the proposed RL can achieve trajectory planning. However, due to the configuration of the planned trajectory points, several unstable points emerge in the middle section of the Bi-RRT planned trajectory, while the RL method can attain smooth trajectory planning. In terms of convergence, Bi-RRT methods exhibit rapid convergence towards feasible solutions at the expense of requiring multiple adjustments. In contrast, RL methods do not necessitate this step, but require numerous training iterations to discover the optimal solution.

**Figure 4:**
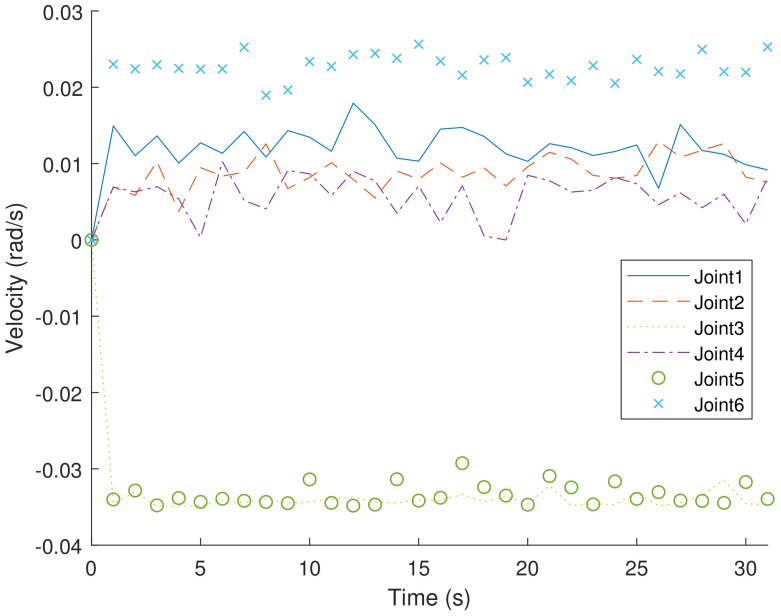
The proposed RL method for time–segment joint velocity optimization in joint angle curves: ($\theta_{1}$, $\theta_{2}$, $\theta_{3}$, $\theta_{4}$, $\theta_{5}$, $\theta_{6}$).

**Figure 5:**
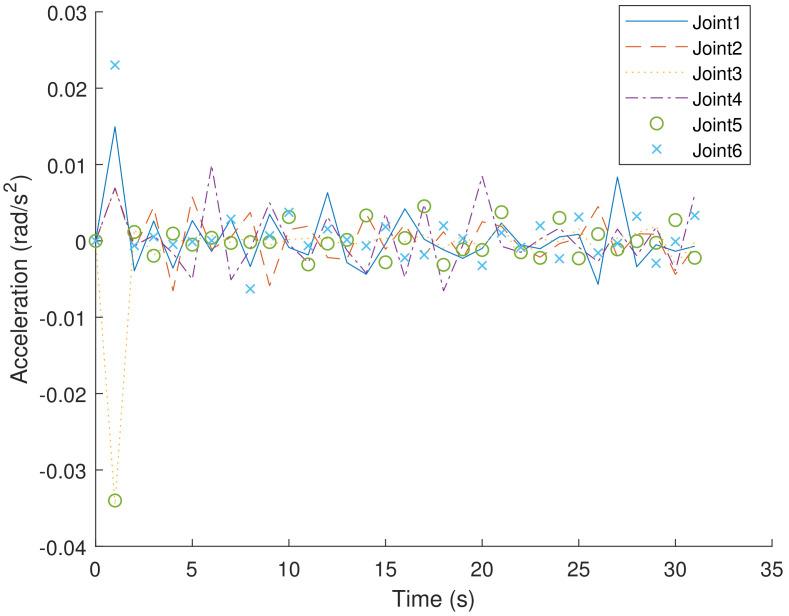
The proposed RL method for time–segment joint acceleration optimization in joint angle curves: ($\theta_{1}$, $\theta_{2}$, $\theta_{3}$, $\theta_{4}$, $\theta_{5}$, $\theta_{6}$).

**Figure 6:**
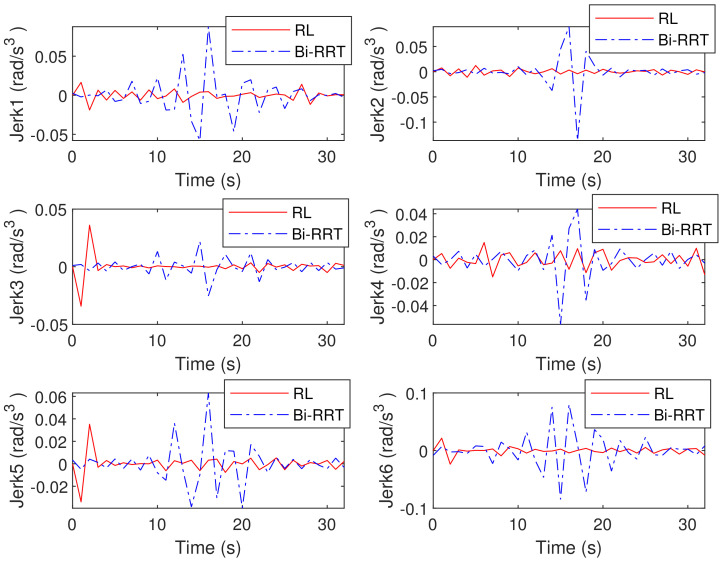
The proposed RL method for time–segment joint jerk minimization in joint angle curves: ($\theta_{1}$, $\theta_{2}$, $\theta_{3}$, $\theta_{4}$, $\theta_{5}$, $\theta_{6}$).

Figure 4 and Figure 5, respectively, show the velocity and acceleration of six joint movements during the trajectory-planning process of the robotic arm. Figure 6 also displays the jerk variation for six joints of the Bi-RRT and RL in the trajectory planning of a six-axis robotic arm. The proposed RL method exhibited a marginally smaller oscillation amplitude compared to the Bi-RRT method, further substantiating that the RL method can optimize the planned trajectory, while the Bi-RRT method cannot accomplish such applications.

**Figure 7:**
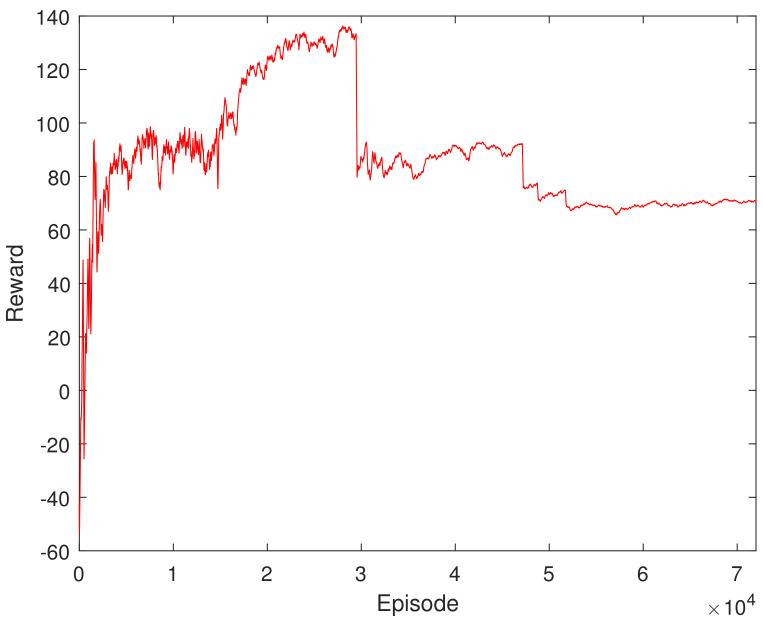
Learning curve of the training process.

**Figure 8:**
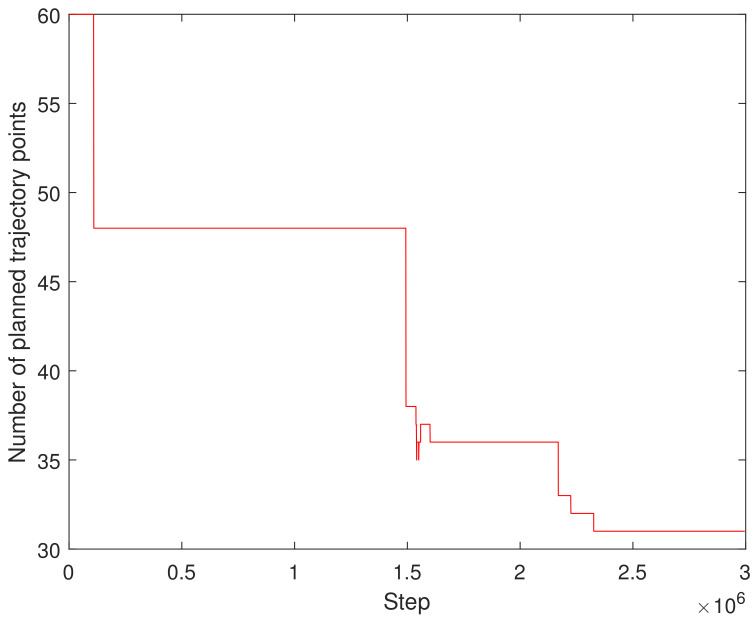
The performance of decaying episode in trajectory planning quantity during training.

Figure 7 depicts the average reward during the training process. It can be seen that the training reward has several stable stages, which is due to the adaptive adjustment of the step time. When the step time changes, the corresponding cumulative reward will undergo new changes and eventually tend to be stable. Figure 8 shows the change in the time step size of the decaying episode throughout the training process. It can be seen from Figure 8 that, as the training continued, the time step of the episode decreased gradually, from 60 to 46, then to 36, and finally, to 32 under the set maximum training conditions, and finally, it tended to be stable. This result can be verified indirectly from Figure 7. This demonstrates the adaptive adjustment of trajectory planning, which corresponds to the continuous updates occurring within the neural network.

### 5.3. Performance Evaluation in Physical Environment

To validate the effectiveness of the proposed RL-based trajectory-planning method in a real-world scenario, a physical experiments was conducted by using a cooperative robotic arm. The experiments were designed to closely follow the simulated scenarios, and the robotic arm’s motion was captured at different time intervals (0 s, 6 s, 12 s, 18 s, 24 s, and 30 s). The performance of the robotic joints’ changes demonstrated the practical applicability of our proposed method.

**Figure 9:**
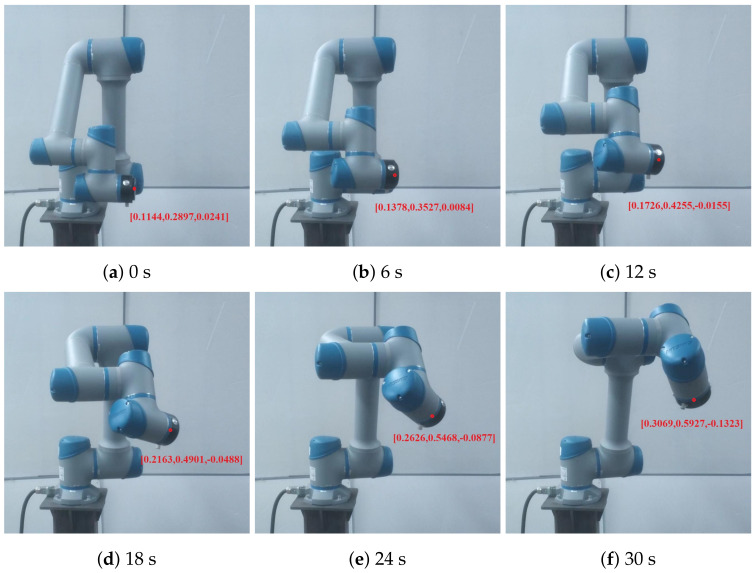
Path–planning visualization for the SIASUN cooperative robot’s movement sequence.

Figure 9 presents the motion of the robotic arm at various time intervals during the experiment. The images reveal that the proposed RL-based trajectory-planning method resulted in smooth and efficient motion, with no sudden jerks or instabilities. The real-world performance of the robotic arm closely matched the simulated results, indicating that the RL method can effectively optimize the planned trajectory, while the Bi-RRT method cannot achieve such performance. In addition to the experimental results detailed in this paper, we have also furnished Supplementary Materials for a more comprehensive understanding. These include a demonstrative video (Video S1), which effectively illustrates the path planning mechanism employed by a six-axis robotic arm.

The physical experiments revealed that the proposed RL-based trajectory-planning method can successfully optimize the robotic arm’s motion in terms of velocity, acceleration, and jerk minimization. This demonstrated the potential of our method for various practical applications, including, but not limited to industrial automation, robotic manipulation, and autonomous systems.

## 6. Conclusions

This study presented a comprehensive investigation into the trajectory-planning problem of a six-axis robotic arm, utilizing deep reinforcement learning to develop a multi-objective optimization approach. This method optimally integrates the goals of minimizing accuracy, energy consumption, and smoothness, allowing for the generation of a desired trajectory for the robotic arm. The input parameters consisted of joint angles and Cartesian coordinates based on forward and inverse kinematics, while the output was the estimation of the joint angles. The decaying episode mechanism was employed throughout the training process, fostering rapid convergence to more-efficient solutions.

Through a comparative analysis with the Bi-RRT algorithm, the proposed method demonstrated its effectiveness and robustness, as evidenced by the simulation results. These findings have the potential to contribute significantly to the future development of advanced robotic arm trajectory planning, enhancing performance, and broadening the applicability of deep reinforcement learning in complex motion-planning tasks.

## Figures and Tables

**Table 1:** M D-H parameters of the 6-axis robotic arm.

Link	$a_{i}$	$\alpha_{i}$	$d_{i}$	$m_{i}$	$I_{\mathrm{ix}}$	$I_{\mathrm{iy}}$	$I_{\mathrm{iz}}$
1	0	$-\frac{\pi }{2}$	0.1055	12.369	0.0696	0.0682	0.0544
2	0	$\frac{\pi }{2}$	0.0969	22.51	0.0939	2.128	2.120
3	0.4181	0	0	6.386	0.104	0.957	0.868
4	0.396	$-\frac{\pi }{2}$	0	3.579	0.0105	0.0062	0.0104
5	0	$\frac{\pi }{2}$	−0.0889	3.579	0.0105	0.0062	0.0104
6	0	$-\frac{\pi }{2}$	0.0845	0.477	0.0007	0.0008	0.0010

**Table 2:** Hyperparameter for reinforcement learning training.

Hyperparameter	Value
Batch size	64
Learning rate	$3\times 10^{-4}$
Clip range	0.2
Discount factor	0.98
Entropy coefficient	$3\times 10^{-3}$
Epoch for updating	10

**Table 3:** The structure of the actor and critic networks.

Layer	Actor	Critic	Activation Function
Input layer	25	25	ReLU
Hidden layer 1	128	128	ReLU
Hidden layer 2	64	64	ReLU
Hidden layer 3	64	64	ReLU
Output layer	6	1	Tanh

## Data Availability

Not applicable.

## Supplementary Materials

The following supporting information can be downloaded at: https://www.mdpi.com/article/10.3390/s23135974/s1, Video S1: The path planning of a six–axis robotic arm.

## Abbreviations

The following abbreviations are used in this manuscript:

RL	Reinforcement learning
PPO	Proximal policy optimization
D-H	Denavit–Hartenberg
RRT	Rapidly-exploring random tree
GAE	Generalized advantage estimation

## References

[1] Tamizi M.G., Yaghoubi M., Najjaran H. (2023). A review of recent trend in motion planning of industrial robots. Int. J. Intell. Robot. Appl..

[2] Liu Y., Guo C., Weng Y. (2019). Online time-optimal trajectory planning for robotic manipulators using adaptive elite genetic algorithm with singularity avoidance. IEEE Access.

[3] Yu X., Dong M., Yin W. (2022). Time-optimal trajectory planning of manipulator with simultaneously searching the optimal path. Comput. Commun..

[4] Liu C., Cao G.H., Qu Y.Y., Cheng Y.M. (2020). An improved PSO algorithm for time-optimal trajectory planning of Delta robot in intelligent packaging. Int. J. Adv. Manuf. Technol..

[5] Wang L., Luo C., Li M., Cai J. (2017). Trajectory planning of an autonomous mobile robot by evolving ant colony system. Int. J. Robot. Autom..

[6] Sudhakara P., Ganapathy V., Sundaran K. Mobile robot trajectory planning using enhanced artificial bee colony optimization algorithm. Proceedings of the 2017 IEEE International Conference on Power, Control, Signals and Instrumentation Engineering (ICPCSI).

[7] Wang T., Xin Z., Miao H., Zhang H., Chen Z., Du Y. (2020). Optimal trajectory planning of grinding robot based on improved whale optimization algorithm. Math. Probl. Eng..

[8] Zhao J., Zhu X., Song T. (2022). Serial manipulator time-jerk optimal trajectory planning based on hybrid iwoa-pso algorithm. IEEE Access.

[9] Singh G., Banga V.K. (2022). Kinematics and trajectory planning analysis based on hybrid optimization algorithms for an industrial robotic manipulators. Soft Comput..

[10] Santos R.R., Rade D.A., da Fonseca I.M. (2022). A machine learning strategy for optimal path planning of space robotic manipulator in on-orbit servicing. Acta Astronaut..

[11] Samir M., Assi C., Sharafeddine S., Ebrahimi D., Ghrayeb A. (2020). Age of information aware trajectory planning of UAVs in intelligent transportation systems: A deep learning approach. IEEE Trans. Veh. Technol..

[12] Bertino A., Bagheri M., Krstić M., Naseradinmousavi P. (2019). Experimental autonomous deep learning-based 3d path planning for a 7-dof robot manipulator. Proceedings of the Dynamic Systems and Control Conference.

[13] Wu Y.H., Yu Z.C., Li C.Y., He M.J., Hua B., Chen Z.M. (2020). Reinforcement learning in dual-arm trajectory planning for a free-floating space robot. Aerosp. Sci. Technol..

[14] Palleschi A., Mengacci R., Angelini F., Caporale D., Pallottino L., De Luca A., Garabini M. (2020). Time-optimal trajectory planning for flexible joint robots. IEEE Robot. Autom. Lett..

[15] Yin S., Ji W., Wang L. (2019). A machine learning based energy efficient trajectory planning approach for industrial robots. Procedia CIRP.

[16] Liu S., Wang Y., Wang X.V., Wang L. (2018). Energy-efficient trajectory planning for an industrial robot using a multi-objective optimisation approach. Procedia Manuf..

[17] Hou R., Niu J., Guo Y., Ren T., Han B., Yu X., Ma Q., Wang J., Qi R. (2022). Multi-objective optimal trajectory planning of customized industrial robot based on reliable dynamic identification for improving control accuracy. Ind. Robot. Int. J. Robot. Res. Appl..

[18] Rout A., Mahanta G.B., Bbvl D., Biswal B.B. (2020). Kinematic and dynamic optimal trajectory planning of industrial robot using improved multi-objective ant lion optimizer. J. Inst. Eng. (India) Ser. C.

[19] Huang J., Hu P., Wu K., Zeng M. (2018). Optimal time-jerk trajectory planning for industrial robots. Mech. Mach. Theory.

[20] Denavit J., Hartenberg R.S. (1955). A kinematic notation for lower-pair mechanisms based on matrices. Trans. ASME J. Appl. Mech..

[21] Cursi F., Bai W., Yeatman E.M., Kormushev P. (2022). GlobDesOpt: A Global Optimization Framework for Optimal Robot Manipulator Design. IEEE Access.

[22] Wang H., Qi H., Xu M., Tang Y., Yao J., Yan X., Li M. Research on the relationship between classic Denavit–Hartenberg and modified Denavit–Hartenberg. Proceedings of the 2014 Seventh International Symposium on Computational Intelligence and Design.

[23] Schulman J., Wolski F., Dhariwal P., Radford A., Klimov O. (2017). Proximal policy optimization algorithms. arXiv.

[24] Kuffner J.J., LaValle S.M. RRT-connect: An efficient approach to single-query path planning. Proceedings of the Proceedings 2000 ICRA. Millennium Conference. IEEE International Conference on Robotics and Automation. Symposia Proceedings (Cat. No. 00CH37065).

